# Effects of the vertically transmitted microsporidian *Facilispora margolisi* and the parasiticide emamectin benzoate on salmon lice (*Lepeophtheirus salmonis*)

**DOI:** 10.1186/s12864-017-4040-8

**Published:** 2017-08-17

**Authors:** Jordan D. Poley, Ben J. G. Sutherland, Mark D. Fast, Ben F. Koop, Simon R. M. Jones

**Affiliations:** 1 Atlantic Veterinary College, University of Prince Edward Island, Department of Pathology & Microbiology, 550 University Ave, Charlottetown, PE C1A 4P3 Canada; 2 Centre for Biomedical Research, Department of Biology, University of Victoria, 3800 Finnerty Rd, Victoria, BC V8W 3N5 Canada; 3 Institut de Biologie Intégrative et des Systèmes (IBIS), Département de biologie, Université Laval, 1030 Avenue de la Medecine, Québec, QC G1V 0A6 Canada; 4 Pacific Biological Station, 3190 Hammond Bay Road, Nanaimo, BC V9T 6N7 Canada

**Keywords:** Copepoda, host-parasite, microsporidia, salmon, sea lice, transcriptomics, vertical transmission

## Abstract

**Background:**

Microsporidia are highly specialized, parasitic fungi that infect a wide range of eukaryotic hosts from all major taxa. Infections cause a variety of damaging effects on host physiology from increased stress to death. The microsporidian *Facilispora margolisi* infects the Pacific salmon louse (*Lepeophtheirus salmonis oncorhynchi*), an economically and ecologically important ectoparasitic copepod that can impact wild and cultured salmonids.

**Results:**

Vertical transmission of *F. margolisi* was demonstrated by using PCR and in situ hybridization to identify and localize microsporidia in female *L. salmonis* and their offspring. Spores and developmental structures of *F. margolisi* were identified in 77% of F_1_ generation copepods derived from infected females while offspring from uninfected females all tested negative for the microsporidia. The transcriptomic response of the salmon louse to *F. margolisi* was profiled at both the copepodid larval stage and the pre-adult stage using microarray technology*.* Infected copepodids differentially expressed 577 transcripts related to stress, ATP generation and structural components of muscle and cuticle. The infection also impacted the response of the copepodid to the parasiticide emamectin benzoate (EMB) at a low dose of 1.0 ppb for 24 h. A set of 48 transcripts putatively involved in feeding and host immunomodulation were up to 8-fold underexpressed in the *F. margolisi* infected copepodids treated with EMB compared with controls or either stressor alone. Additionally, these infected lice treated with EMB also overexpressed 101 transcripts involved in stress resistance and signalling compared to the other groups. In contrast, infected pre-adult lice did not display a stress response, suggesting a decrease in microsporidian virulence associated with lice maturity. Furthermore, copepodid infectivity and moulting was not affected by the microsporidian infection.

**Conclusions:**

This study demonstrated that *F. margolisi* is transmitted vertically between salmon louse generations and that biological impacts of infection differ depending on the stage of the copepod host. The infection caused significant perturbations of larval transcriptomes and therefore must be considered in future studies in which impacts to host development and environmental factors are assessed. Fitness impacts are probably minor, although the interaction between pesticide exposure and microsporidian infection merits further study.

**Electronic supplementary material:**

The online version of this article (doi:10.1186/s12864-017-4040-8) contains supplementary material, which is available to authorized users.

## Background

Microsporidia are a diverse group of obligate intracellular, spore-forming parasites of invertebrates and vertebrates with over 150 genera and 1200 recognized species [1]. Molecular phylogenetic evidence indicates that microsporidia are highly specialized fungi that parasitize a wide range of hosts [2]. Many microsporidia have damaging impacts in agriculture, apiculture, and aquaculture [3–5], as well as in human health [6] particularly among the immunocompromised [1]. Microsporidia are also of interest due to their use as biological control agents against insect pests [7, 8]. To date, microsporidia infecting humans and important insects have received the most research attention despite nearly half of all known microsporidia having aquatic hosts [9]. For example, microsporidian infections of crustaceans, a diverse subphylum with approximately 67,000 species [10, 11], are only beginning to be uncovered. Given the ubiquity of crustaceans in the aquatic environment, the lack of information on interacting stressors within these organisms, and the economic importance of crustacean culture (i.e. 6.9 M tonnes worth $36 billion USD; [12]), studies examining the consequences of microsporidian infections are needed.

The microsporidian spore is the only stage capable of surviving outside of a host cell [1] and is the infective stage. Spores contain sporoplasm (i.e. infectious cytoplasm) and a polar filament that erupts under pressure and penetrates the host cell, providing a route through which the sporoplasm and nucleolar material can enter. Merogonic development of the parasite enlarges the host cell and this is followed by sporogonic development of the parasite. Spores can be released to infect adjacent cells (i.e. autoinfection), or to infect other individuals (e.g. through urine, faeces, decomposition) [1]. Passage of infective spores among individual hosts in water or food may be the most common mode of horizontal transmission. Spores can also transmit vertically through eggs to infect offspring. Given the intimate association of microsporidia within host cells, it is not surprising that these parasites can have major effects on their hosts [13]. Drastic host transcriptomic reductions in various functions have been observed in vivo [14]. Pathological and physiological consequences of microsporidia infections have been characterized for a few terrestrial species, but those of infections in the marine environment remain poorly understood.

Microsporidia have evolved reduced genomes and other biological components (e.g. the known genome sizes are in the range of bacteria, 2.3–19.5 Mb) [1]. The genome of *Encephalitozoon cuniculi* is well characterised and consists of ~2.9 Mb across 11 chromosomes, with approximately 2000 potential protein coding genes [15]. Most studied microsporidia have a conserved set of microsporidia-specific genes, suggesting that genome reduction may have occurred prior to the diversification of the lineage [16, 17], but lineage-specific gene expansion and novelty is expected. The molecular basis and mechanisms of host manipulation are still being uncovered (recently reviewed in [18]).

Salmon lice are ectoparasitic copepods belonging to the family Caligidae (suborder Siphonostomatoida) that infect both wild and farmed salmonids, causing losses of more than $500 M USD/annum globally to the aquaculture industry [19]. The salmon louse (*Lepeophtheirus salmonis*) is the most well studied species and has a direct life cycle with two free-living naupliar stages, an infective copepodid stage, two sessile parasitic chalimus stages, and three motile parasitic preadult and adult stages during which the lice are larger and more damaging to the host [20]. Genetically distinct subspecies of *L. salmonis* occur in the Atlantic and Pacific Oceans [21, 22]. Infections on farmed Atlantic salmon (*Salmo salar*) are controlled by treatment with in-feed emamectin benzoate (EMB), although lice in the Atlantic Ocean have become resistant to this drug and to many others (reviewed by [23]). Alternative, non-chemical management options are required, and given the relevance of microsporidia to pest control combined with recent descriptions of microsporidia infections in sea lice, this area requires investigation.

Infections with two microsporidian species have been described in sea lice. *Desmozoon lepeophtherii* was originally identified in *L. salmonis* infecting farmed *S. salar* in Scotland and Norway [24–26] and a genetic variant of *D. lepeophtherii* was described from *L. salmonis* on farmed *S. salar* in the Pacific Ocean [27, 28]. The second microsporidian, *Facilispora margolisi*, was identified in *L. salmonis* infecting *S. salar* and Pacific salmon (*Oncorhynchus* spp.), and in two other *Lepeophtheirus* species from salmonid and non-salmonid fishes in the northeast Pacific Ocean [28]. The prevalence of *D. lepeophtherii* in Pacific *L. salmonis* ranges from 5 to 15% whereas that of *F. margolisi* ranges from 50 to 90% [28].

Histological evidence of *F. margolisi* spores within ovarian tissue [28] suggests the possibility of vertical transmission. Furthermore, nothing is known regarding the effects of the infection on the survival, infectivity and fecundity of *L. salmonis*. Here we provide further evidence of vertical transmission of *F. margolisi* and determine whether the microsporidian affects copepodid survival and infectivity. We then characterize the impacts of the microsporidian infection on the copepod transcriptome, compare this response to known stress genes for *L. salmonis*, and determine whether the response is affected by the addition of a stressor (low dose EMB). Finally, we use this dataset and identify probes that are probably of microsporidian origin on the commonly used *L. salmonis* microarray. We then use these probes to diagnose infection status in an existing published transcriptome dataset of pre-adult *L. salmonis*, validate the findings using RT-qPCR, and compare the responses of pre-adult and copepodid lice to the microsporidian infection.

## Results

### Vertical transmission of *F. margolisi*

Four experiments (Exp.) were conducted to evaluate different aspects of the biology of *F. margolisi*, including vertical transmission in *L. salmonis* (Table 1). Exp. 1 was used to evaluate vertical transmission of the microsporidian. Exp. 2 was also for this purpose, but included an experimental infection to test the effect on infectivity and development. Exp. 3 was used for in situ hybridization in order to determine locations of *F. margolisi* within the louse. Exp. 4 was used to perform transcriptome profiling of the interaction of *F. margolisi* infection and EMB exposure. Further details regarding each experiment are provided in Table 1. *F. margolisi* was detected in the cephalothorax and egg string samples of approximately 60% of the screened F_0_ individuals within these four experiments (*n* = 124). The microsporidian was detected in both the cephalothorax and matched egg string from 96% (*n* = 47) of *F margolisi* positive (MS+) copepods in which both tissues were tested. In the first experiment (Exp. 1), when individual egg strings were used to cultivate F_1_ individuals, 92% of the MS+ egg strings gave MS+ copepodid pools and none of the MS- egg strings produced MS+ offspring.

**Table 1:** Microsporidia (MS) *Facilispora margolisi* infections in adult female salmon lice *Lepeophtheirus salmonis* (F_0_) and their F_1_ larval progeny as determined by PCR

EXP	Source of F_0_ lice	F_0_ individual (MS+ / total)		F_1_ samples (MS+ / total)	
		Cephalothorax	Egg String	F_0_ positive	F_0_ negative
1	Chum Salmon	12/20	12/20	11/12 (pools)	0/8
2	Chum Salmon	14/24	13/24	28/39	0/42
3	Atlantic Salmon	25/40	N/A	N/A	N/A
4	Atlantic Salmon	23/40	22/40	2/2 (pools)	0/2
Total		74/124 (60%)	47/84 (56%)	28/39 (72%)^a^	0/42 (0%)^a^

In experiment (Exp.) 1, each F_1_ nauplius and copepodid pool originated from a single F_0_ egg string. In Exp. 2, F_1_ chalimus II staged lice had developed from pools of MS+ or MS- eggs derived from F_0_ females. In Exp. 3, F_1_ nauplius and copepodid pools were reserved for in situ hybridizations. In Exp. 4, 40 larval cultures derived from MS+ or MS- F_0_ females were divided into 16 pools for each of MS+ and MS- groups, 14 for microarray analysis and 2 for PCR confirmation of *F. margolisi* (PCR confirmation pools shown in the table). All PCR-positive results in the egg string were also positive in the cephalothorax. ^a^Totals only include individuals, not pools

At 15 days post infection, Chum Salmon (*Oncorhynchus keta*) were infected with *L. salmonis* copepodids of known MS infection status (Exp. 2, Table 1) and there was no significant difference in the intensity of infection for MS+ or MS- lice (MS+: 6.5 ± 0.56; MS-: 7.0 ± 0.52 lice / fish; Chi^2^-test, *p* = 0.53). Most copepods in both infection groups (97.4% and 95.2%, respectively) had developed to the late chalimus II stage at the time of sampling. *F. margolisi* was detected in 72% of the chalimus II lice derived from MS+ egg strings but not in any from MS- egg strings (see Exp. 2 in Table 1).

### Histological identification of *F. margolisi* in *L. salmonis*

**Fig. 1:**
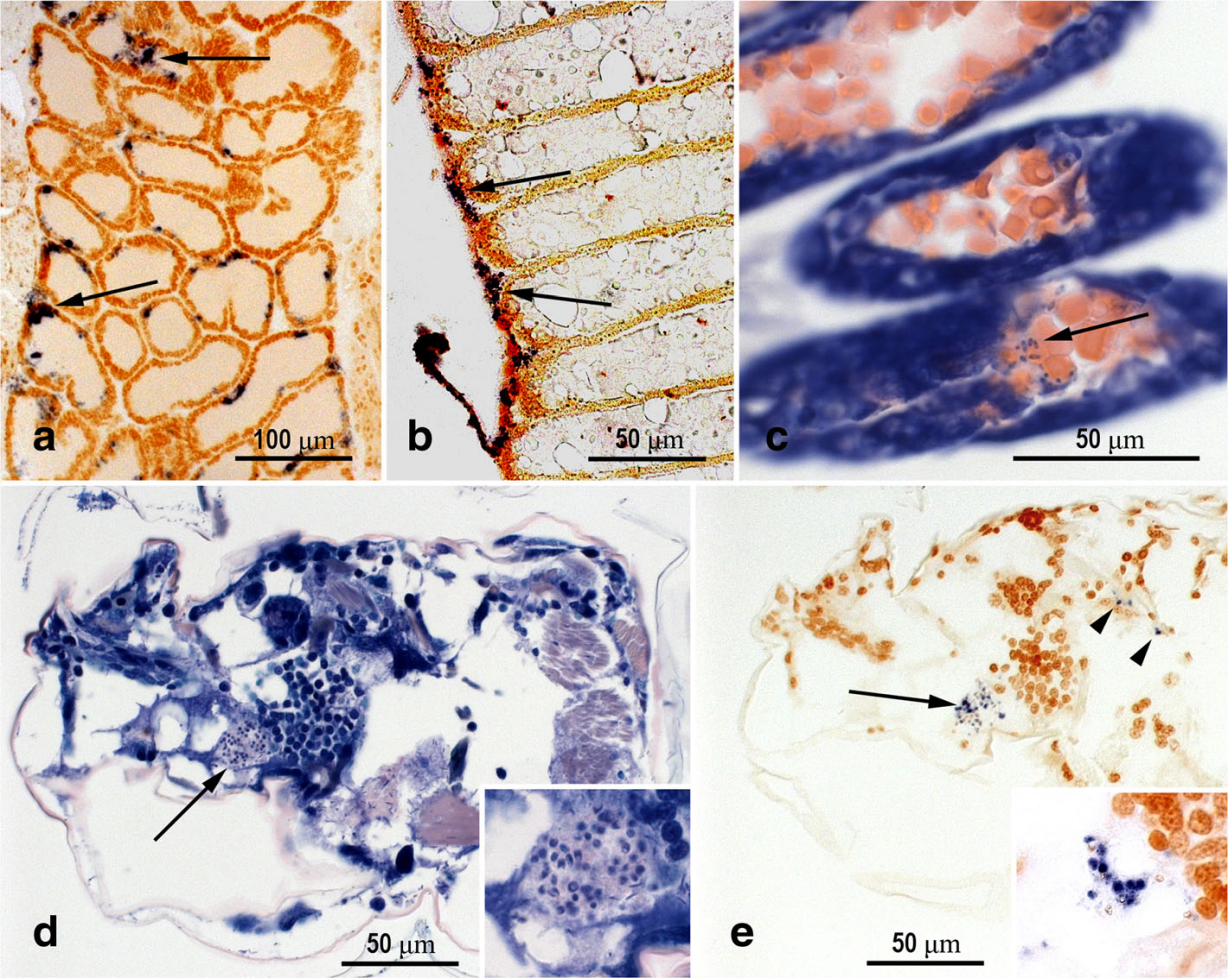
Vertical transmission of *Facilispora margolisi* in *Lepeophtheirus salmonis*. Microsporidian infection (*arrows*) in the copepod ovary (**a**) and egg string (**b**) by in-situ hybridisation. Microsporidian spores (*arrow*) in Giemsa-stained egg string (**c**). Microsporidian spores (*arrows* and *arrowheads*) in Giemsa-stained (**d**) and in-situ hybridisation (**e**) preparations of nauplius larvae. Insets are higher magnification images

In-situ hybridization (ISH) identified *F. margolisi* DNA in histological sections from adult female *L. salmonis* shown previously to be PCR-positive for *F. margolisi* (see Exp. 3). In the adult louse, ISH-positive spores and non-sporogonic structures were associated with cells immediately below the cuticle, in striated muscle, glandular tissues, ovary and egg strings (Fig. 1). ISH-positive reactions were also visualised within unidentified tissues of larval copepods derived from PCR-positive females (Fig. 1). No ISH reactions were observed in PCR-positive samples incubated with unlabelled probe or in PCR-negative samples incubated with the labelled probe.

### Transcriptomic response of copepodid salmon lice to F. margolisi

#### Overview

**Fig. 2:**
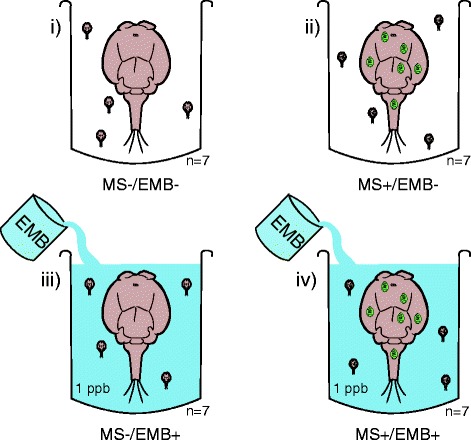
Experimental design for the transcriptome response of copepodid lice to emamectin benzoate (EMB) or microsporidia (MS). Each condition has seven biological replicates and each replicate is a pool of copepodid lice. The factorial design allows for analysis of genes responding to MS, EMB or having an interaction effect of the two factors

**Fig. 3:**
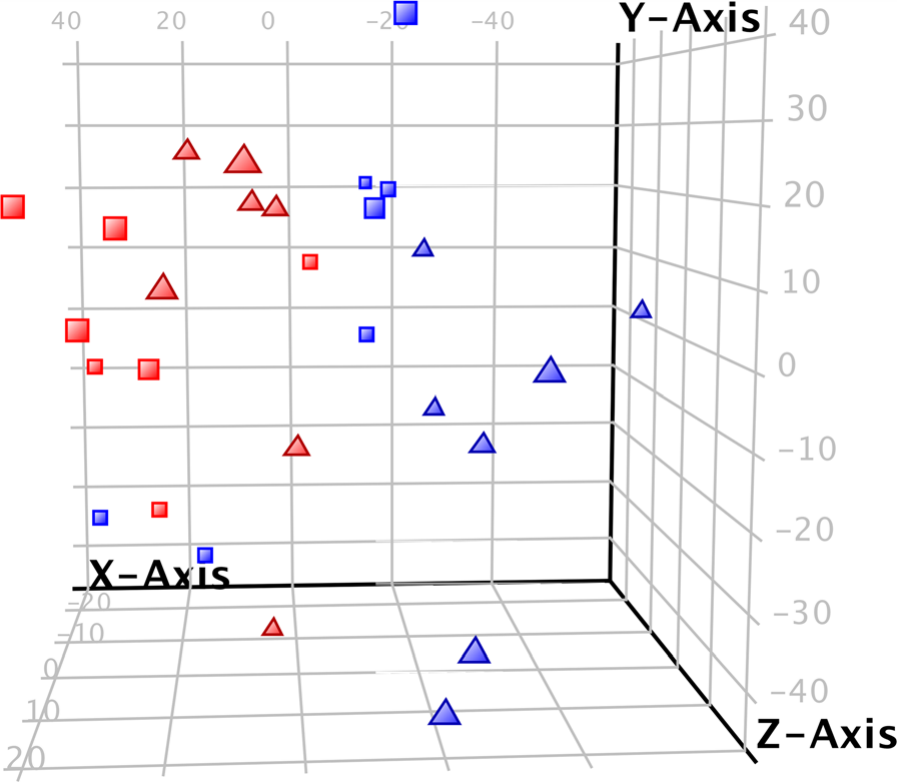
Principal Component Analysis of copepodid samples clustered by gene expression. Samples are quality filtered transcriptome profiles of lice pools that are either positive (*blue*) or negative (*red*) for the microsporidia infection. Clustering indicates that PC1 (X-axis) explains the most variation, with 29.5% of total variation explained. Samples were either exposed to the parasiticide emamectin benzoate (*triangles*) or control (*square*), but this had less of an effect than that of the microsporidia. PC2 (Y-axis) explains 24.5% and PC3 (Z-axis) explains 7.1% of the total variation. PC2 and PC3 were not as clearly associated with a treatment group as was the infection status, which was separated along PC1

**Fig. 4:**
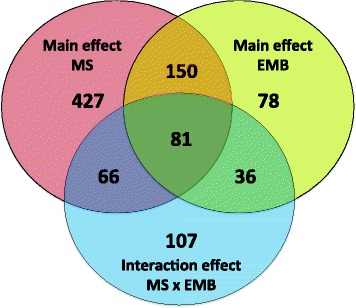
Significantly differentially expressed transcripts (2-way ANOVA; *p* < 0.01 and FC ≥ 1.5) in copepodids in response to the microsporidia *F. margolisi*, to the parasiticide EMB, or an interaction of the two

Copepodid pools (F_1_; see Exp. 4), each originating from a single adult female (MS+ or MS-), were incubated either in seawater alone (unexposed control) or in seawater containing 1 ppb emamectin benzoate (EMB) to evaluate the effect of MS infection and the effect of combined MS and EMB on the *L. salmonis* transcriptome (7 pools per condition, 28 pools in total; Fig. 2). Principal components analysis (PCA) of all quality filtered transcripts indicated a larger effect of MS than of EMB (Fig. 3). The first axis differentiated the MS+ and MS- pools and explained 29.5% of the total variation. Clustering associated with EMB exposure was less obvious. Treatment groupings most distant from one another in the PCA were MS−/EMB- and MS+/EMB+. The number of differentially expressed transcripts also supported a greater effect of MS than 1 ppb EMB (Fig. 4). MS infection affected 577 transcripts whereas EMB affected 228 transcripts (main effects with no interaction effect; *p* ≤ 0.01 and fold change (FC) ≥ 1.5; Additional file 1). The range of FC was also greater for transcripts with a main effect of MS (FC = −9.7 to 4.5) compared with those showing a main effect of EMB (FC = −2.8 to 2.4; Additional file 1). Similarities between the effects MS and EMB were indicated from 150 transcripts concordantly differentially expressed by EMB and MS (Fig. 4; Additional file 1).

To better characterize response genes, transcripts were classified into the following categories: i) MS-specific response genes; ii) general stress response genes, responding concordantly to MS and EMB; and iii) interacting stressor genes, whose response depended on the presence of the second stressor (see Fig. 4). As the majority of transcripts affected by EMB exposure also responded to MS infection (Fig. 4), an EMB-specific response category was not included. All transcripts with an EMB-specific response can be found in Additional file 1.

#### MS-specific response genes

Of the 577 transcripts responding to *F. margolisi* infection (main effect, no MS/EMB interaction effect), 427 (74%) were MS-specific (Fig. 4). Of these, 212 were overexpressed and 215 were underexpressed in MS+ *L. salmonis*.

Overexpressed MS-specific transcripts included 12 cuticle-associated transcripts (e.g. *cuticle protein 6*, *cuticle protein 7*, *cuticle protein C14.6*) and 18 transcripts associated with muscle (e.g. *myosin heavy chain muscle*, *myosin-3*, *tropomyosin*, *tropomyosin-2*, *tropomyosin alpha-1 chain*, *troponin I* and *troponin T*; Additional file 1). Cytoskeleton-related transcripts were overexpressed and enriched (GO: cytoskeleton, 14 transcripts; *p* = 0.003) in MS+ lice including *restin homolog*, *actin clone 403*, *actin-related protein 2/3 complex subunit 4*, *actin cytoplasmic 1*, *tubulin alpha-1A chain*, *tubulin alpha-3 chain*, and *tubulin beta-1A chain*.

Underexpressed MS-specific transcripts were related to the mitochondria (e.g. GO: mitochondrial part, 16 transcripts; *p* < 0.01; Additional file 2) and ribosomes. Mitochondria-related transcripts included *NADH dehydrogenase 1 alpha subcomplex subunits 2, 10* and *12*, *cytochrome c oxidase subunit 1*, *5B* and *6B*, *CYP450 2 J2* and *CYP450 2 L1* (Additional file 1). Ribosomal proteins (rp) underexpressed in MS+ lice included those present in the mitochondria (e.g. *28S rp s32*, *39S rp L22*, *39S rp L23*, *39S rp L32*, *40S rp s12* and *60S rp L12*) and those in the cytoplasm (e.g. *40S rp s16*, *60S rp L3*, *60S rp L29*, *60S rp L30*, *60S rp L44*, *rp s6 kinase beta 2*), suggesting an overall reduction in ribosome constituents in the MS+ lice.

#### General stress response genes

A total of 150 transcripts were overexpressed in both the MS+ and EMB+ treatment groups (main effects, no interaction effect). These were enriched for protein folding and ATP binding functions (Table 2; Additional file 2). Overexpressed transcripts included several previously associated with stress in *L. salmonis* such as *60 kDa heat shock protein mitochondrial*, *DnaJ homolog subfamily A member 1*, *heat shock 70 kDa protein cognate 4*, *heat shock protein 90-alpha*, *heat shock cognate protein 90-beta* and T-complex proteins (TCP) *TCP-1-alpha*, *TCP-1-beta*, *TCP-1-epsilon*, *TCP-1-theta*, and *TCP-1-zeta* (Additional file 1; [29]). Fold changes of these transcripts were more modest than those of MS-specific response genes, and were typically in the range of 1.5- to 2.1-fold (Additional file 1).

**Table 2:** Unique SwissProt IDs for transcripts differentially expressed by EMB exposure and *F. margolisi* infection

Functional annotation term	Accession ID	Count (# Unique SwissProt IDs)			*P*-value of enrichment	
		MS	EMB	Shared	MS	EMB
Overexpressed contigs						
ATP binding	GO:0005524	25	18	10	4E-04	2E-05
Chaperone	Keywords	16	10	10	2E-08	3E-06
Chaperonin-containing T-complex	GO:0005832	4	4	4	3E-04	5E-05
Chaperonin Cpn60/TCP-1	IPR002423	9	6	6	3E-11	2E-07
Cytosol	GO:0005829	15	11	7	0.002	0.001
Nucleotide-binding	Keywords	31	17	9	2E-05	8E-04
Protein folding	GO:0006457	17	11	11	6E-10	3E-07
Protein metabolic process	GO:0019538	36	26	15	4E-04	1E-05
Underexpressed contigs						
Disulfide bond	Keywords	20	12	9	2E-05	8E-05
Extracellular region	GO:0005576	17	8	7	1E-05	0.003
Glycoprotein	Keywords	21	15	13	0.005	1E-04
Integral to membrane	GO:0016021	32	17	14	0.005	0.005
Peptidase activity, acting on L-amino acid peptides	GO:0070011	13	8	7	7E-04	0.002
Peptidase S1 and S6, chymotrypsin/Hap	IPR001254	6	4	3	6E-04	0.004
Protease	Keywords	13	8	7	8E-04	0.002
Serine protease	Keywords	8	6	5	8E-05	9E-05
Secreted	Keywords	13	8	7	1E-04	5E-04
Substrate-specific transmembrane transporter activity	GO:0022891	10	6	5	0.02	0.03
Transmembrane	Keywords	32	17	14	3E-04	0.001

Transcripts (unique SwissProt IDs only) from each main effect list (2-way ANOVA; *p* < 0.01; FC ≥ 1.5 main effect and no interaction effect) were analyzed independently and then compared for similar functional enrichments including Gene Ontology (GO), InterPro (IPR), and SP_PIR_Keyword (Keywords; [61–63]. The numbers of transcripts responding to both stressors are displayed in the shared count column. See Additional files 1 and 2 for transcript IDs and SwissProt accessions for each functional term

Underexpressed general stress response transcripts included seven proteases (e.g. *trypsin-1*, *neprilysin-11*, *carboxypeptidase B*, and *hypodermin-B*; Table 2; Additional file 1). All underexpressed proteases were annotated as secreted by SP_PIR_Keyword, and this list was enriched for peptidase activity acting on L-amino acid peptides (Table 2). Furthermore, multiple solute carrier (SLC) family members were underexpressed including *high affinity copper uptake protein 1* (*slc31A1*), *solute carrier family 15 member 1* (*slc15A1*) and *sodium-dependent phosphate transport protein 2B* (*slc34A2*). These transcripts, and others, enriched the GO category substrate-specific transmembrane transporter activity (Table 2). Transporters were underexpressed in MS+ lice and in EMB+ lice, and five of these were underexpressed in both groups.

#### Interacting stressor response genes

**Fig. 5:**
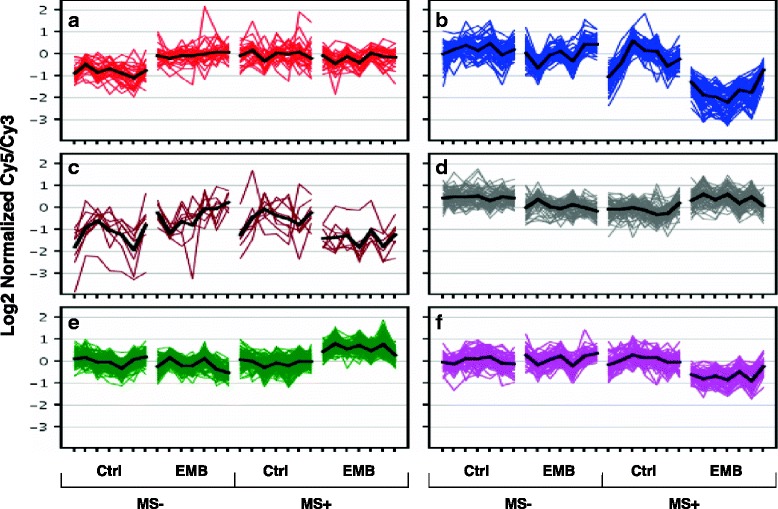
Stressor interacting genes clustered by expression profiles. Transcripts (*n* = 290) responding to MS or EMB differently depending on the presence of the second stressor (i.e. significant interaction effect) as characterized by *k*-means clustering of expression levels in six clusters (A-E; *k* = 6). Mean expression profiles are represented by the *black lines* for each cluster

The expression of 290 transcripts showed a significant interaction between EMB exposure and MS (*p* < 0.01 and FC ≥ 1.5; Fig. 4). The expression similarities among these transcripts were characterized by *k*-means clustering to separate them into six general patterns (clusters A-F; Fig. 5). Transcripts in clusters A, C, and E were overexpressed and B, D, and F were under-expressed in at least one of the treatment groups relative to the controls.

The majority of the transcripts with significant interaction effects belong to two classes. Genes in cluster A (32/290 transcripts; Fig. 5) had equal overexpression in both single stressors (MS or EMB) but were not overexpressed additively in the double stressor condition (MS+/EMB+). These transcripts were similar to the general stress response genes described above, including *calreticulin*, *Dnaj homolog subfamily B member 11*, and *stress-induced-phosphoprotein 1* (Additional file 3). The second main class included transcripts in clusters E (101/290) and F (49/290), that were only affected in the double stressor group (EMB+/MS+) (Fig. 5). Cluster E included overexpressed *catalase*, *DnaJ homolog subfamily A member 1*, *heat shock protein 81–1*, or structural transcripts such as *myosin heavy chain*, *myosin light chain alkili*, *muscle M-line assembly protein unc-89*, *tropomyosin*, and many others (Additional file 3). Cluster F included transcripts only underexpressed in MS+/EMB+ lice such as the transporters *slc25A36*, *protein spinster*, and *RhGB*. *RhGB* was previously shown to be down-regulated in *L. salmonis* by EMB, cypermethrin, and hyposalinity [30].

**Fig. 6:**
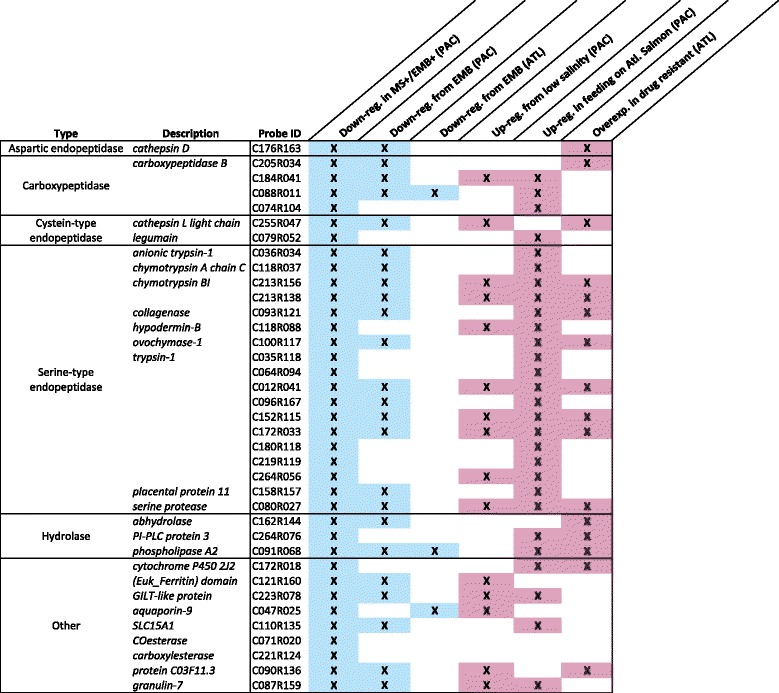
Multiple experiment analysis of double stressor down-regulated genes. The 48 transcripts in Cluster B were underexpressed in all EMB+/MS+ lice compared to other groups (interaction effect, *p* < 0.01 and FC ≥ 1.5). Using these transcripts, a cross-experiment comparison was performed with previously published *L. salmonis* transcriptome data, which indicated that these transcripts may be involved in feeding, stress responses, and drug resistance. Only annotated transcripts are shown. Further information on the transcripts in Cluster B and the studies with similar transcripts can be found in Additional file 4

Cluster B had the largest fold changes, and contained 48 transcripts 1.8- to 8-fold underexpressed in the double stressor condition (Fig. 5). Some of these transcripts were also slightly downregulated (FC < 2) in MS+ lice. Cluster B largely contained serine-type endopeptidases including those annotated as *trypsin-1* (9 different contigs), *anionic trypsin-1*, *chymotrypsin A chain C*, *chymotrypsin BI* (2 different contigs), *hypodermin-B*, *ovochymase-1*, and *collagenase* (Fig. 6; Additional file 3). Also included were other degradative enzymes such as *cathepsin-L light chain*, *cathepsin-D*, and *carboxylesterase* (2 different contigs), and detoxification-related transcripts such as *cytochrome p450 2 J2* and *carboxypeptidase B* (4 different contigs). Of the 34 annotated transcripts in Cluster B, 22 were annotated with the SP_PIR_Keyword secreted, 16 of which contained at least one trypsin-like protease domain (CDD: smart00020, NCBI).

Transcripts within cluster B were further compared with other published *L. salmonis* microarray datasets due to their putative secreted degradative enzyme function. Of the 48 unique transcripts (based on unique contig identifiers), 36 were overexpressed in actively feeding lice relative to starved lice (Fig. 6; [31]), 20 transcripts were overexpressed in EMB-resistant relative to EMB-sensitive Atlantic lice, and 32 transcripts were down-regulated by 50 ppb EMB exposure in pre-adult Pacific lice [32]. Furthermore, 20 transcripts were affected in Pacific copepodids by hyposaline conditions (Fig. 6 and Additional file 4) [29]. These genes may therefore be important for a range of processes, including feeding, stress response and drug resistance.

### Microsporidian genes on the salmon louse microarray

Archived individual transcriptome profiles of pre-adult Pacific salmon lice exposed to very low concentrations of EMB that did not affect the transcriptome (GEO accession: GSE73734; [33]) were tested for the presence of *F. margolisi* using PCR probes (see Methods). Of the 40 samples, 22 (55%) were MS+ and 18 were MS-, permitting the analysis of genes affected by MS within these pre-adult individuals.

In the pre-adult transcriptomes, few differences were observed between MS+ and MS- individuals (Additional file 5). However, the expression of 20 transcripts was detected in all 22 MS+ individuals, but not in any of the 18 MS- samples (i.e. 100% of MS- samples had a Cy5 value below the QC filter background). Interestingly, these transcripts did not pass background fluorescence thresholds in any of the copepodid samples from the previously described MS/EMB study, regardless of MS infection status. Of the 20 transcripts expressed exclusively in MS+ pre-adults, 14 (70%) were annotated to genes from other microsporidian parasites such as *Spraguea lophii* (4 transcripts) and *Nosema ceranae* (3 transcripts; Table 3). Two of the remaining six transcripts were annotated to the slime mold *Dictyostelium discoideum* and the causative agent of malaria *Plasmodium falciparum*, and four had no annotation. The presence and absence of expression in the MS+ and MS- samples identified on the microarray was validated using RT-qPCR for *subunit alpha of phenylalanyl-tRNA synthetase*, *heat shock protein 90*, and 40*S ribosomal protein S4* (Table 3; Additional file 6).

**Table 3:** Transcripts expressed exclusively in MS+ pre-adult *L. salmonis*, putatively of microsporidia *F. margolisi* origin

Contig ID	SwissProt	E-value	Description	Organism
5725753^a^	S7XG51	4E-76	*Ribosomal S4*	*Spraguea lophii*
5733902^a^	S7XQV3	6E-84	*Beta-tubulin*	*Spraguea lophii*
5722217^a^	A0A0F9Z835	5E-67	*Heat shock 90*	*Nosema ceranae*
5734902^a^	H8Z9X9	3E-56	*Heat shock 90*	*Nematocida parisii*
5725884^a^	A0A0F9WFB6	2E-60	*Heat shock 70*	*Nosema ceranae*
5724134^a^	A0A0B2UJK9	3E-132	*Phenylalanyl-tRNA synthetase alpha*	*Ordospora colligata*
5729536^a^	A0A059EU36	9E-129	*Ribosomal S4*	*Anncaliia algerae*
5727693^a^	L7JXU0	0	*Elongation factor 2*	*Trachipleistophora hominis*
5723341^a^	Q25002	1E-153	*Elongation factor 1*	*Glugea plecoglossi*
5727856^a^	S7XVR1	7E-75	*Heat shock 70*	*Spraguea lophii*
5726266^a^	A0A0F9WBW4	2E-116	*Tubulin alpha*	*Nosema ceranae*
5722879^a^	S7XU13	7E-155	*Diphosphate reductase*	*Spraguea lophii*
5,727,653	Q553P2	3E-11	*Uncharacterized*	*Dictyostelium discoideum*
5734574^a^	C8CG41	4E-9	*Polar tube protein*	*Antonospora locustae*
5,724,095	W4J6D6	5E-9	*Uncharacterized*	*Plasmodium falciparum*
5733921^a^	E0S8Y5	4E-14	*Uncharacterized*	*Encephalitozoon intestinalis*

Annotation using e < 10E-5 with UniProt BLASTx [68]

^a^Contig sequence best aligns with another microsporidian sequence

The exclusive presence of these transcripts in MS+ individuals, combined with their annotation to genes from other microsporidian species suggests that they are not from *L. salmonis* but rather of microsporidia origin in the original samples used to create the microarray. The concordance in profiles between the diagnostic primers [28], microarray probes, and RT-qPCR primers suggests that these sequences may be useable to screen for the presence of *F. margolisi* in individual adult *L. salmonis* samples, but not in copepodid pools. These probes will be flagged as microsporidian origin in future updates of the 38 k microarray annotation file.

## Discussion

Microsporidia can have large effects on their hosts and have potential as biological control agents in pest management. In this study, we demonstrate that *Facilispora margolisi*, a parasite of *L. salmonis* in the Eastern Pacific Ocean [28], is vertically transmitted and has stage-specific impacts on host energetic and structural gene expression. Infection was associated with transcriptomic evidence of a stress response in copepodids, which was absent in the pre-adults. However, infection with *F. margolisi* did not affect the number of *L. salmonis* copepodids able to attach and moult on Chum Salmon. Combinations of drug and MS infection impacted transcripts previously identified as related to feeding and drug response and resistance. These results are discussed below in terms of the evolution and biology of key traits in salmon lice.

### Microsporidia transmission and stage-specific effects

Vertical transmission of microsporidia is common in other crustacean hosts and can be the sole means of transmission [34] or as part of a mixed transmission strategy [35]. Vertical transmission of *F. margolisi* was identified using PCR and in situ hybridization to locate the parasite within the ovary and developing embryos of infected *L. salmonis* females and their offspring. Spore dimorphism in *F. margolisi*, specifically the presence of larger spores with longer polar filaments, indicates the possibility of horizontal transmission [28], but this was not yet confirmed experimentally. Vertically transmitted microsporidia generally are less virulent than horizontally transmitted species as they depend on host survival for replication [36]. Nonetheless, vertically transmitted MS can affect host growth [34], and induce male feminization [37], thereby impacting sex ratios [38].

Despite the absence of measurable effects on *L. salmonis* infectivity or development, infection with *F. margolisi* impacted host transcriptomes particularly in larval stages. MS+ copepodids differentially expressed 577 transcripts that were enriched for energetic and stress functions whereas in adults only 123 transcripts were differentially expressed. Of these transcripts, only five were differentially expressed in both developmental stages, including a mitochondrial chaperone *DnaJ homolog subfamily A member 1*, *calreticulin*, and three transcripts without annotation (Additional files 1 and 5). The reason for the reduced host impact at later lice life stages is not known, but it may be related to the reduced virulence required for vertical transmission. Furthermore, copepodids are generally more sensitive to stress than adults. For example, pre-adult *L. salmonis* show a transcriptional response to 50 ppb EMB but not 25 or 10 ppb EMB [32] while copepodids respond at 1 ppb EMB, as observed here. Improved understanding of the sites of microsporidian infections during early copepod development may inform our understanding of the physiological impacts of these infections.

### *Lice response to* F. margolisi

MS+ copepodids underexpressed mitochondrial component genes, including ribosomal subunits and those involved in mitochondrial organization. Close proximity between early stages of *F. margolisi* and mitochondria of *L. salmonis* has been observed [28]. This is a common strategy among microsporidia that results from reduced mitosomes and an inability to generate ATP [2]. Intricate manipulations of the host mitochondria structure, and function permit energy acquisition for microsporidian growth and development [39, 40]. In the host this can cause prolonged energetic stress by up-regulating important metabolic pathways [41–43]. Increased metabolic activity was suggested in this study as MS+ copepodids overexpressed genes involved in cellular respiration and ATP binding while transcripts related to mitochondrial structure were underexpressed. It is therefore possible that *F. margolisi* is associated with manipulation of mitochondrial structure and function in *L. salmonis*.

The overexpression of muscle- and cuticle-related transcripts in *F. margolisi* infected copepodids may also be a result of manipulation by the microsporidia. Mature spores and other developmental stages of *F. margolisi* were observed within striated muscle and subcuticular connective tissue of *L. salmonis* using in situ hybridization, supporting previous findings from electron microscopy [28]. Although there was no indication of an innate immune response against *F. margolisi* in the lice, this may be due to the generally poor annotation of immunity genes in crustaceans [44]. Microsporidian infections are known to impact a variety of immune-related genes in other arthropods [45, 46] and nematodes [47]. Transcripts without annotation accounted for 47% of those affected by MS and may be useful to consider for future studies on biotic stress in *L. salmonis*.

### Non-additive impacts on lice hosts from parasiticide and microsporidia

Our observed interactions between drug treatment and microsporidian infection may be important in understanding the development of drug resistance in *L. salmonis*. In this study, differences from controls in the transcriptome of MS+ *L. salmonis* exposed to EMB were greater than differences from either stressor alone, and a total of 290 transcripts showed differential expression in a non-additive manner. In the western honey bee *Apis mellifera*, additive impacts were observed between MS infection and chemical exposure that resulted in changes to fitness costs depending on the mechanism of drug resistance [48]. Pesticide exposure may also affect microsporidian virulence and host susceptibility, as in *Flabelliforma magnivora* and *Nosema ceranae* infecting *Daphnia magna* [49] and *A. mellifera* [50], respectively.

The largest fold changes between MS+/EMB+ lice and controls were for transcripts in cluster B (Fig. 5), which were underexpressed in only the MS+/EMB+ lice. These specific transcripts are also involved in feeding [31], stress response [29], and EMB resistance [32]. Braden et al. [31] found 36 of the 48 cluster B transcripts to be underexpressed after 24 h and 48 h of starvation (Additional file 4). Based on annotation, these transcripts may be involved in feeding and digestion, as well as host immunosuppression (Fig. 6) and included 16 trypsin-like serine proteases. Trypsin-like serine proteases are present in lice secretions [51–53] and many of these transcripts were most highly expressed in the gut relative to other tissues [54]. Another transcript from this cluster, *hypodermin B*, inhibits the activation of complement component C3 *in vitro* [55] and may therefore play a role in immunomodulating salmonid hosts. Although *F. margolisi* infection or EMB exposure alone had minimal effects on these transcripts, in combination they resulted in underexpression (FC = 1.5–8.0). Future studies should address the localized effects of *F. margolisi* infection based on large gene expression changes associated with infected lice tissues (i.e. muscle, cuticle, mitochondria, and glandular tissue) (Fig. 1 and [28]) in order to determine possible interactive effects of microsporidia infection and louse feeding, digestion, mobility, moulting, energy expenditure, and overall fitness.

In addition, 32 of the 48 cluster B transcripts were also found to be down-regulated from an exposure to 50 ppb EMB in Pacific pre-adult lice, and overexpressed in an EMB resistant Atlantic *L. salmonis* population (Additional file 4; [32]). Resistance to EMB occurs in *L. salmonis* in the Northeast and Northwest Atlantic Ocean while lice in the Pacific subspecies remain sensitive [23, 56]. Although there is no evidence that the presence of *F. margolisi* contributes to the maintenance of EMB sensitivity in the Pacific lice, the correlation and the non-additive impacts of *F. margolisi* and EMB on genes involved in stress, feeding, and host attachment indicate the need for further research to better understand a role of microsporidian infections on drug tolerance, selection, and fitness.

### Non-host material in next-generation sequencing data

We identified a set of 20 transcripts within the *L. salmonis* microarray that most likely belong to the microsporidian *F. margolisi*. The presence of non-host material in a sequence database highlights the importance of identifying and/or removing non-target eukaryotic sequences from assemblies, as wild individuals or lines with persisting, vertically transmitted intracellular eukaryotic parasites represent a mixture of organisms [57]. These microsporidian-like transcripts were only detectable in cDNA generated from MS+ pre-adults, but not in that from MS+ copepodids, potentially indicating stage-specific expression of *F. margolisi* genes or a higher effective microsporidian load in pre-adult lice. Future RNA-sequencing studies can potentially use these genes to quantify microsporidian infections in *L. salmonis* as was done with microsporidia of humans [14].

## Conclusions

Although *F. margolisi* induced a stress-like transcriptomic response in copepodids of *L. salmonis*, signatures of stress were absent in pre-adults and the infection did not cause changes in salmon lice development or infection potential towards fish hosts. Collectively our observations of limited impacts within the later developmental stages are consistent with vertical transmission of the microsporidian and its high prevalence within *L. salmonis* populations. However, the non-additive effects from combinations of microsporidia infection and parasiticide treatment merit further study. In addition to microsporidia, the salmon louse also hosts viruses [58] and bacteria [59] and the present observations indicate a need to better understand the possible influence of hyperparasitism with respect to copepod sensitivity to parasiticides as well as the confounding effects these infections may have on transcriptomic studies.

## Methods

### Animals

Ovigerous *Lepeophtheirus salmonis* were collected from wild adult Chum Salmon (*Oncorhynchus keta*) captured in a regulatory test fishery or from farmed Atlantic Salmon (*Salmo salar*) during harvest and maintained in aerated seawater at 10 °C for no longer than 48 h before processing.

Chum Salmon from the Nanaimo River Hatchery were reared from swim-up fry in a mixture of freshwater and seawater (9.2 °C – 10.0 °C) and provided a daily ration of commercial pellets.

### Culture of *L. salmonis* larvae

Four experiments (Exp. 1–4) were conducted in which the *F. margolisi* infection status of individual adult female *L. salmonis* and their nauplius or copepodid offspring were determined and compared (Table 1). For each experiment between 20 and 40 ovigerous copepods bearing two intact and pigmented egg strings were selected. From each, one egg string was placed into a sterile flask containing 300 mL of aerated seawater and incubated at 10 °C for 6 to 8 days. The second egg string and the anterior third of the cephalothorax were preserved separately in 95% ethanol and the remainder of the specimen was preserved in neutral buffered 10% formalin (NBF). All dissections were conducted using tools rinsed sequentially with 4% sodium hypochlorite, water and 95% ethanol between specimens. The infection status of each specimen was determined from separate polymerase chain reactions (PCR, *see below*) of the cephalothorax and egg-string.

In Exp. 1, 20 larval cultures were separately filtered through 47 mm cellulose acetate/cellulose nitrate membranes with a pore size of 8.0 μm (EMD Millipore). The membranes were flash-frozen in liquid nitrogen and stored at −80 °C for subsequent PCR analysis. In Exp. 2, 24 larval cultures were pooled according to infection status of the source copepod. The numbers of copepodids and nauplii in each pool were determined by microscopic examination and used to infect naïve salmon (*see below*). In Exp. 3, 40 larval cultures were separately pooled according to infection status of the source copepod and fixed in NBF for 24 h followed by storage in 70% ethanol for in situ hybridisation assays (*see below*). In Exp. 4, 40 larval cultures were pooled according to the *F. margolisi* infection status of the source copepod. Each pool was divided into 16 sub-pools, 14 of which were maintained in aerated seawater for use in the emamectin benzoate (EMB) exposure study (*see below*) while the remaining two pools from each of infected and uninfected females were filtered and stored as above for PCR confirmation.

### Polymerase chain reaction (PCR) detection

DNA was extracted from ethanol-preserved specimens using the DNeasy Animal Tissue protocol (Qiagen), as per manufacturer’s instructions. A region of *F. margolisi* SSU rDNA was amplified with PCR as described by [28]. Samples were scored *F. margolisi* positive (MS+) or negative (MS-) using 1.5% agarose gel electrophoresis.

### Exposure of salmon to *L. salmonis*

Juvenile Chum Salmon (11.0 ± 0.3 g; *n* = 6 per tank) were acclimated for 6 days in duplicate 30-L tanks containing aerated seawater at 30 ppt and 9 °C, flowing at 1 L min^−1^. Fish in each tank were exposed to 1570 and 1680 7-day post hatch (dph) copepodids derived from infected and uninfected female copepods (Exp. 2, above), respectively, using the method described earlier [60]. At 15 dph, the salmon were euthanized by immersion in 200 mg L^−1^ tricaine methane sulphonate and all copepods were removed and stored in 95% ethanol. Each copepod was assessed microscopically for stage of development and for the presence of *F. margolisi* by using PCR.

### Histology

NBF-preserved specimens from Exp. 3 were processed routinely for histology and subsequent microscopic examination as previously described [28]. During processing the larvae were aggregated prior to embedding to facilitate detection. Sections of 5 μm were applied to uncoated or silane coated glass microscope slides and stained routinely (Gram or Giemsa stains), or processed for in-situ hybridisation, respectively.

### *In-situ* hybridization probe design, synthesis and assay

An 84 bp region of the *F. margolisi* SSU rRNA gene was amplified (see primers in Additional file 6) to serve as template for subsequent digoxygenin (DIG) labelling reactions using a probe synthesis kit (Roche Applied Science). Each reaction contained 2 μl of genomic DNA, 1× PCR reaction buffer (Invitrogen), 1.5 mM MgCl2 (Invitrogen), 0.2 mM of each dNTP, 0.025 U/μl of Platinum® *Taq* DNA polymerase (Invitrogen), and sterilized water. Positive (*F. margolisi* DNA) and negative (water) controls were included alongside all reactions. The PCR profile consisted of 95 °C for 5 min, 35 cycles at 95 °C for 30 s, 55 °C for 30 s, and extension at 72 °C for 2 min, followed by a final extension at 72 °C for 10 min. Amplified PCR products were visualized on ethidium bromide stained, 1% agarose gels. PCR products were purified using QIAquick® PCR Purification Kit (Qiagen) and quantified with NanoDrop-1000 Spectrophotometer. DIG-labelling reactions were carried out according to the manufacturer’s instructions using 30 ng of purified PCR product with the same primers and thermal profile as the conventional PCR and DIG labelled products were purified as described above.

The ISH protocol was adapted from earlier work (Antonio et al. 1998, Jones et al. 2003). Tissue sections on silane-coated slides were routinely deparaffinized and permeabilized with 20 ng/μl of proteinase K (Qiagen) for 10 min. Sections were incubated with 50 μl of hybridization buffer (5.1 ml deionised formamide, 2.0 ml 20× standard saline citrate (SSC; 3 M NaCl, 0.3 M sodium citrate, pH 7.0), 2.0 ml 5% dextran sulphate, 0.5 ml denatured sperm DNA (10 mg/ml), 0.2 ml 50× Denhardt’s solution (1% acetylated BSA, 1% polyvinylpyrrolidone, and 1% Ficoll 400 in molecular biology grade water) and 25 μl 10% SDS per 10 ml of solution) for 1 h at 37 °C. A volume of 50 μl of digoxygenin (DIG) - labelled probe (0.1 ng/μl in hybridization buffer) was placed on each tissue section. Hybridization, formazan precipitation, and probe visualization were performed at 37 °C following an established protocol [61]. Controls included sections from PCR-negative copepods stained with DIG - labelled probe and sections from PCR-positive copepods stained with unlabelled probe.

### Exposure to EMB and extraction of RNA

Four experimental conditions were tested (Exp. 4) based on the presence of the microsporidian *F. margolisi* (MS+ or MS-) and EMB (EMB+ or EMB-). These conditions are labeled as MS+/EMB+, MS+/EMB-, MS−/EMB+, MS−/EMB-, and each with seven biological replicates. Each replicate was a flask containing between 50 and 75 copepodids. EMB (Sigma-Aldrich) was dissolved at a concentration of 1.0 ppb and aerated cultures were maintained for 24 h at 10 °C before filtration, flash-freezing and storage as above. The frozen filters were homogenized (mixer mill; Retsch® MM 301), and total RNA was extracted using TRIzol® (Invitrogen), as per manufacturers’ instructions. Total RNA was purified through RNeasy spin columns with an on-column DNase I treatment (Qiagen). Total RNA was quantified by spectrophotometry (NanoDrop-1000), and quality checked by electrophoresis on a 1% agarose gel. Samples were then randomized for all downstream nucleic acid manipulations.

### Microarray Analysis

Labeled cRNA was generated from total purified RNA using Low Input Quick Amp Labeling kits (Agilent) as per manufactures’ instructions and as reported previously [29]. A Cy3-cRNA pool was generated for a reference design by synthesizing Cy3-cRNA from three randomly selected samples from each of the four experimental conditions and combining equimolar amounts from each into a common pool. Samples were hybridized as per manufacturer’s instructions as previously described [29] to a 38 K oligo microarray designed using previously annotated ESTs from both Pacific and Atlantic *L. salmonis* [62] using eArray (Agilent). Slides were scanned on a ScanArray® Express (Perkin Elmer) at 5 μm resolution using PMT settings optimized to have the median signal of ~1–2% of array spots saturated (Cy5: 70; Cy3: 75).

Images were quantified in Imagene 8.1 (Biodiscovery), poor spots flagged, and background corrected as reported previously [29]. Sample files were loaded into GeneSpring 13.0 (Agilent). Raw sample files have been uploaded to GEO (GSE94692). Samples were normalized as follows: raw value threshold of 1.0; intensity-dependent Lowess normalization; and baseline transformation to the median of all samples. Control spots, and any probes not passing the following filter were removed from the analysis: raw values ≥500 in both Cy3 and Cy5 channels and no poor quality flags in at least 65% of samples in any one condition. A principle component analysis was performed within GeneSpring (Agilent) on the samples to investigate for any clustering of technical or biological variables.

Probes were tested using a two-way ANOVA (*p* < 0.01; FC ≥ 1.5) for main effects of microsporidia, main effects of EMB, and a significant interaction effect of microsporidia and EMB. Probes with a significant interaction effect were subtracted from the main effect lists to be considered with the interaction effect list. Probes with a significant interaction effect were separated by *k*-means clustering using the least number of clusters until cluster redundancy was visible (Euclidean distance metric; 6 clusters; 50 iterations; Agilent). Gene Ontology and pathway analysis was performed on significant entity lists using UniProt accession numbers in the DAVID bioinformatics tool [63–65] using a modified Fisher’s exact test (*p* < 0.05; genes/enrichment category ≥4) with a background list of all entities passing quality control filters. This background list was used for a principal components analysis that clustered samples according to expression of genes. Samples were colored or shaped differently in order to identify trends associated with experimental conditions.

### Pre-adult *L. salmonis* exposed to EMB

A fifth experiment, which was previously described [32], included exposure of F1 generation pre-adult II *L. salmonis* (21 females and 19 males) to either seawater, 0.01 ppb EMB (Sigma-Aldrich), or 0.1 ppb EMB. These lice were collected from farmed Atlantic salmon in the Broughton Archipelago, British Columbia (BC). Using the previously extracted RNA, as described [33], cDNA for detection of the microsporidian and qPCR of selected genes in these archived samples was synthesized for individual lice from 1 μg of purified RNA using iScript™ Reverse Transcription Supermix kit (BioRad) as per manufacturer’s instructions (see [32]). Detection of *F. margolisi* was completed as described earlier [28] with the exception that cDNA derived from purified RNA was used as template and a touch-down PCR assay with decreasing annealing temperatures from 63 °C to 58 °C was completed before standard PCR for an additional 25 cycles to amplify the target sequence.

The microarray results were validated using RT-qPCR of five targets to confirm expression patterns. Primer efficiencies were confirmed by creating standard curves with 5–6 points and a 5-fold series dilution with a r^2^ > 0.95 and effeciencies between 0.90 and 1.05. For RT-qPCR amplification, SsoAdvanced SYBR Green Supermix (BioRad) was used in 11 μl reactions with 1 μl template and 0.1 μM of each primer using the following program: 95 °C for 2 min, followed by 40 cycles of 95 °C for 5 s and 60 °C for 15 s. Melt curve analysis was performed at the end of each qPCR assay by increasing the temperature in 0.5 °C increments (65 °C to 95 °C) every 5 s. No template controls (NTC) and no RT controls were negative for all genes assayed. Normalization for genes of interest was completed using qbase-PLUS (Biogazelle; Gent, Belgium) with an output of log_2_ ratios relative to the reference genes *elongation factor 1α* (*ef1α*) and *ribosomal protein subunit 20* (*rps20*). Reference gene stability was assessed using geNorm [66], which showed an M value of 0.35 and a coefficient of variation (CV) of 0.13.

Normalized log_2_ expression measured by RT-qPCR was compared to Cy5/Cy3 log_2_ expression ratios produced by the microarray. For genes specifically expressed in MS+ lice on the microarray, this was validated by RT-qPCR. For genes expressed in all conditions, a Pearson’s correlation (R v. 3; [67]) used to validate the microarray (Additional file 6).

## Additional files

Additional file 1: Differentially expressed transcripts by microsporidia and EMB. (XLSX 244 kb)

Additional file 2: Gene Ontology. (XLSX 30 kb)

Additional file 3: Interaction of microsporidia and EMB within *k*-means clustering. (XLSX 95 kb)

Additional file 4: Consensus transcripts within Cluster B across *L. salmonis* studies. (XLSX 107 kb)

Additional file 5: Pre-adult *L. salmonis* responses to *F. margolisi* infection and microsporidian genes on the microarray. (XLSX 85 kb)

Additional file 6: Primers for RT-qPCR validation and *in situ* hybridization. (XLSX 10 kb)

## Abbreviations

ANOVA: Analysis of variance
ATP: Adenosine triphosphate
bp: Base pairs
dph: Days post hatch
EMB: Emamectin benzoate
F_1_: First generation
GO: Gene ontology
ISH: In situ hybridization
MS: Microsporidian *F. margolisi*
NBF: Neutral buffered formalin
PCA: Principal components analysis
PCR: Polymerase chain reaction
RNA: Ribonucleic acid
SSU rRNA: Small subunit ribosomal RNA
